# CD8^+^ T cells from vitiligo perilesional margins induce autologous melanocyte apoptosis

**DOI:** 10.3892/mmr.2012.1117

**Published:** 2012-10-08

**Authors:** JILONG WU, MIAONI ZHOU, YINSHENG WAN, AIE XU

**Affiliations:** 1 Department of Dermatology, The Third People’s Hospital of Hangzhou, Hangzhou 310009, P.R. China; 2 Department of Biology, Providence College, Providence, RI 02918, USA

**Keywords:** CD8^+^ T cells, melanocytes, vitiligo, cytokine

## Abstract

Cell-mediated autoimmunity has been suggested to be involved in the melanocyte apoptosis that occurs in vitiligo. We investigated the cytotoxicity to autologous melanocytes of CD8^+^ T cells from the perilesional margins and peripheral blood samples of vitiligo patients. CD8^+^ T cells isolated from skin biopsied from the edges of depigmented skin patches of vitiligo patients or from peripheral blood samples of the same donors were proliferated in culture medium. The primary cultures of CD8^+^ T cells and autologous melanocytes were mixed at ratios of 1:1, 1:2 or 1:5 and incubated for 3 days. The apoptosis of the melanocytes was analyzed by flow cytometry. Secreted cytokines in selected samples were measured by cytokine arrays. The results show that the CD8^+^ T cells were successfully isolated from the vitiligo perilesional margins. This cell population showed a significantly higher percentage of CD69 expression (56.13±3.55 versus 29.93±2.35%, p<0.01) and CD137 expression (41.74±1.06 versus 25.97±1.63%, p<0.01) compared with CD8^+^ T cells in peripheral blood from the same donors. The co-culturing of CD8^+^ T cells from lesional skin with autologous melanocytes induced apoptosis in the melanocytes (16.63±1.21, 16.71±0.63 and 18.32±1.60% for CD8^+^ T cells and autologous melanocytes at ratios of 1:1, 1:2 and 1:5, respectively). IL-6 levels were much higher in the co-culture (3.01-fold higher than in a melanocyte monoculture and 17.32-fold higher than in a CD8^+^ T-cell monoculture). The CD8^+^ T cells were also demonstrated to secrete more IL-13. Taken together, our data demonstrate that the infiltration of active CD8^+^ T cells takes place in the vitiligo perilesional margins. Those CD8^+^ T cells present significantly higher activation levels and higher cytotoxicity to autologous melanocytes than their counterparts from peripheral blood samples. These data suggest that CD8^+^ T cells are likely to be involved in the pathogenesis of vitiligo.

## Introduction

Vitiligo is a skin disorder with no fatality but significant psychosocial consequences. It is characterized by the progressive depigmentation of sections of skin. While the causes of this disease remain to be determined, various factors have been suggested to be involved in the pathogenesis. Cytokines and reactive oxygen species play important roles in the disappearance of melanocytes. Further evidence has pointed to the involvement of cellular immunity (1).

Studies revealing that melanocyte-specific antibodies (2) and cytotoxic T cells (3) have been detected in the peripheral blood of vitiligo patients supports the autoimmune hypothesis. The cytotoxicity of CD8^+^ T cells to melanocytes is considered to make a key contribution to the pathogenesis of non-segmental vitiligo (4). The autoimmune response is suggested to trigger the vitiligo but is not the main factor that affects the extent and duration of the disease (5–7).

It has been reported that T-cell numbers in the peripheral blood samples of vitiligo patients are normal, while the ratio of CD4^+^/CD8^+^ decreases and the NK cell number either increases or is unchanged. An immunohistological study of cutaneous lymphocytes in vitiligo revealed that infiltration of CD8^+^ T cells occurs surrounding the vitiligo lesions (8–12).

In normal skin, similar amounts of CD4^+^ and CD8^+^ T cells are found in the dermis (13,14). T cells on the leading edge of the vitiligo lesion are homing lymphocytes, which specifically recognize autologous melanocytes (11,12,15). Understanding the relationship between CD8^+^ T-cell infiltration of the perilesional margin and the loss of melanocytes in lesional skin should shed light on the possible role of the autoimmune response in vitiligo pathogenesis.

Given the possibility of the involvement of cellular immunity in the pathogenesis of vitiligo, we undertook this study to investigate the potential effects of CD8^+^ T cells from vitiligo perilesional margins on the apoptosis of autologous melanocytes using a co-culture system. Our results support the theory that CD8^+^ T cells from the perilesional region of the vitiligo-affected skin induce autologous melanocyte apoptosis leading to the disappearance of melanocytes.

## Materials and methods

### Reagents

RPMI-1640 was purchased from Sijiqing Biological Engineering Materials Co., Ltd. (Hangzhou, China). Fetal bovine serum (FBS), recombinant human IL-2 and IL-15, Dynabeads^®^ Human T-Expander CD3/CD28 and Dead Cell Apoptosis kit with Annexin V Alexa Fluor^®^ 488 and propidium iodide (PI) were obtained from Life Technologies Corporation (Grand Island, NY, USA). PerCP anti-human CD3 antibody, PE anti-human CD8a antibody and the corresponding anti-human IgG antibodies were acquired from BioLegend, Inc. (San Diego, CA, USA). FITC anti-CD69, anti-CD137 and anti-IgG antibodies were obtained from BD Biosciences (San Jose, CA, USA). The lymphocyte separation buffer was supplied by Sigma (St. Louis, MO, USA). The CD8^+^ T Cell Isolation kit was obtained from Miltenyi Biotech (Auburn, CA, USA). The RayBio^®^ Human Cytokine Antibody array was purchased from RayBiotech, Inc. (Norcross, GA, USA) and data analysis was performed by Shanghai KangCheng Biotech (Shanghai, China).

### Patients

Patients were recruited from the Department of Dermatology, The Third People’s Hospital of Hangzhou, China. All participants gave written informed consent. Skin biopsies were obtained by surgery from patients with stable vitiligo who had failed to respond to autologous epidermis or melanocyte transplantation at least twice. Peripheral blood samples were extracted from the same patients. Systemic application of glucocorticoid and immunosuppressive drugs was restricted for one month prior to the surgery.

### Isolation and amplification of CD8^+^ T cells

A donor skin specimen, 0.5 × 1.0 cm in size, was obtained from the perilesional margin of a depigmented patch. Subcutaneous tissue was removed from the skin. The remaining skin was washed with culture medium (RPMI-1640 with 10% fetal calf serum, 5 ng/ml IL-15, 2 ng/ml IL-2 and 1% streptomycin-ampicillin) three times and then cut into small sections (0.1–0.2 cm). The sections of skin were placed in 12-well plates and cultured in 1 ml medium together with 1.25 μl CD3/CD28 antibody-coated Dynabeads for 4–5 days. Subsequently, half of the medium was changed every 2–3 days. Sufficient lymphocytes for further experiments were obtained after 3 weeks.

### Characterization of CD8^+^ T cells

The primary lymphocyte culture was centrifuged at 1,000 rpm for 10 min. The resulting cell pellet was washed with PBS twice and re-suspended in 0.1 ml PBS. Either 5 μl PerCP anti-human CD3 antibody or 20 μl PE anti-human CD8a antibody was added to the cells which were then incubated at 4°C for 30 min. Expression levels of CD3 and CD8a were analyzed by flow cytometry. Mouse IgG was used as a control.

### Determination of activation level of CD8^+^ T cells

Following the labeling of 0.1 ml CD8^+^ T cells (1×10^6^/ml) with 20 μl FITC anti-CD69 or PE anti-CD137 antibodies, the expression levels of CD69 and CD137 were examined by flow cytometry.

### Isolation of CD8^+^ T cells from blood samples

Intravenous blood (5 ml) was extracted from the vitiligo patients. Peripheral blood mononuclear cells were separated by the Ficoll/Hypaque density gradient centrifugation method and washed twice with PBS. The CD8^+^ T Cell Isolation kit was then used to purify the CD8^+^ T lymphocytes.

### Apoptosis analysis

Melanocytes were isolated and cultured by methods based on those described previously (16,17). Primary melanocytes (passage 3–5) from vitiligo patients or normal controls were seeded into 6-well plates at a density of 1×10^5^ cells/well and kept in an incubator for 4 h to allow them to attach. CD8^+^ T lymphocytes isolated from the perilesional margin or peripheral blood of the same patient were then added to the wells at a ratio of 1:1, 2:1 or 5:1. After 72 h of co-culturing, the supernatant with lymphocytes was removed and the melanocytes were stained with PI and Annexin V. The apoptosis rate was then analyzed by flow cytometry.

### Cytokine array assay

CD8^+^ T cells from the perilesional margin were co-cultured with autologous melanocytes. Monocultures of CD8^+^ T cells or melanocytes were prepared in parallel as controls. Supernatants were collected after 3 days and analyzed for cytokine level by cytokine array.

## Results

### Primary culture and characterization of CD8^+^ T lymphocytes

To investigate the effect of CD8^+^ T lymphocytes on the apoptosis of melanocytes, we first isolated these cells from the perilesional skin and peripheral blood of the vitiligo patients. T lymphocytes were isolated from skin surrounding the depigmented patches. Up to 10^7^ T cells were prepared after 3 weeks of culture and the success rate was >95%. It was confirmed by flow cytometry that >90% of this T-cell population were CD8-positive. CD8^+^ T cells were also isolated from peripheral blood of the same donors. A total of 92.05% of prepared cells were confirmed to be CD8-positive.

### CD8^+^ T cells from different sources have different activation levels

**Figure 1:**
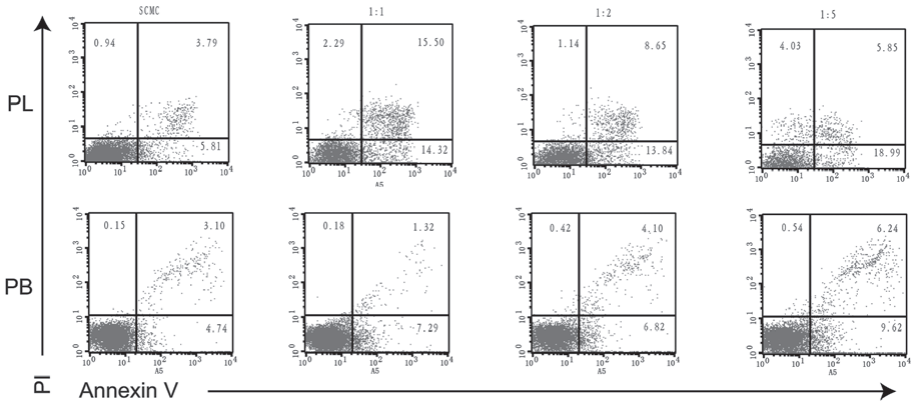
Melanocyte apoptosis induced by various ratios of CD8^+^ T cells. PB, peripheral blood CD8^+^ T cells; PL, perilesional CD8^+^ T cells.

Next we tested the activation levels of the CD8^+^ T cells from the two sources. The CD8^+^ T cells isolated from peripheral blood or perilesional skin were examined by flow cytometry to determine the expression levels of CD69 and CD137, which are indicators of T-cell activation. As shown in Table I, the CD8^+^ T cells from perilesional skin have much higher levels of CD69 (56.13±3.55 versus 29.93±2.35%, p<0.01) and CD137 (41.74±1.06 versus 25.97±1.63%, p<0.01) than CD8^+^ T cells from peripheral blood. These data indicate that CD8^+^ T cells from the perilesional region are more active (Fig. 1).

### CD8^+^ T cells from vitiligo lesions show higher capacity to induce apoptosis in co-cultured autologous melanocytes

**Figure 2:**
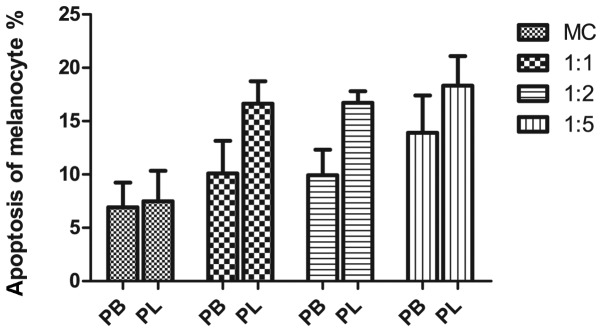
Comparison of melanocyte apoptosis induced by various amounts of CD8^+^ T cells. MC, melanocytes; PB, peripheral blood CD8^+^ T cells; PL, perilesional CD8^+^ T cells.

To further investigate the effect of the CD8^+^ T cells on autologous melanocytes, we co-cultured the cells. The results revealed the substantial apoptosis of melanocytes when purified CD8^+^ T cells were co-cultured with autologous melanocytes (Fig. 2). Melanocytes cultured alone had an apoptotic rate of 6.92±1.34%; the percentage of apoptosis increased to 10.10±1.76, 9.93±1.39 and 13.90±2.03% when the melanocytes were co-cultured with CD8^+^ T cells from peripheral blood in ratios of 1:1, 1:2 and 1:5, respectively. The apoptotic rate of melanocytes increased from 7.49±1.65 to 16.63±1.21, 16.71±0.63 and 18.32±1.60% when co-cultured with CD8^+^ T cells from the perilesional margin at ratios of 1:1, 1:2 and 1:5, respectively. While CD8^+^ T cells from both sources induced apoptosis in autologous melanocytes, those from the vitiligo lesion demonstrated significantly higher cytotoxicity.

### CD8^+^ T cells from perilesional skin and peripheral blood show different cytokine profiles

**Figure 3:**
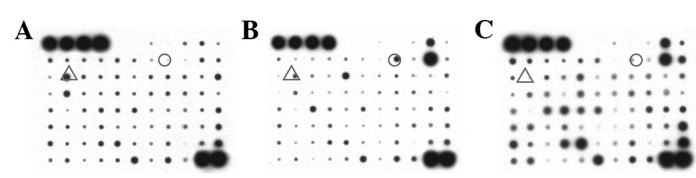
Cytokine array of the culture supernatants. (A) CD8^+^ T cells; (B) CD8^+^ T cells and melanocytes; (c) melanocytes. In the co-culture, IL-6 levels were much higher and the CD8^+^ T cells secreted more IL-13. The IL-6 level in (B) was 3.01-fold that in (C) and 17.32-fold that in (A) (dots marked as ○ in the figure); the IL-13 level in (B) was 8.02-fold that in (C) and 0.56-fold that in (A) (dots marked as △).

Cytokines are important in cytotoxicity; therefore, we measured the cytokine profile for the co-culture system. Cytokine array analysis (Fig. 3) demonstrated that the level of IL-6 in the supernatant of the perilesional CD8^+^ T cell/autologous melanocyte co-culture was 3.01-fold higher than that in the melanocyte monoculture (IL-6 standard value: 0.403 versus 0.134) and 17.32-fold higher than that in the peripheral blood CD8^+^ T cell/melanocyte co-culture (0.403 versus 0.023). IL-13 levels in the supernatant of the lesional CD8^+^ T cell/autologous melanocyte co-culture were also increased 8.02-fold compared with the melanocyte monoculture (0.321 versus 0.040) but decreased compared with the blood CD8^+^ T cell/melanocyte co-culture (0.321 versus 0.719, 0.56-fold).

## Discussion

The *in situ* activities of perilesional T cells in the effector phase of depigmentation were analyzed in a skin explant model by van den Boorn *et al* who found that T cells infiltrating the perilesional margin specifically recognize melanocyte antigen and are reactive to melanocyte antigen-specific stimulation. These T cells were able to selectively induce the apoptosis of melanocytes when they infiltrated autologous normally pigmented skin explants (15).

Our study demonstrated that perilesional CD8^+^ T cells express relatively high levels of CD69 and CD137, the surface markers of activated T cells. The percentage of CD69 expression in the perilesional CD8^+^ T-cell population was determined to be 56.13±3.55%, while that in their peripheral blood counterparts was 29.93±2.35%. The percentage of CD137 expression in the perilesional CD8^+^ T cells was 41.74±1.06%, which is also higher than that in the peripheral blood CD8^+^ T cells (25.97±1.63%, p<0.01). These results suggest that the perilesional CD8^+^ T cells are more highly activated than the peripheral blood CD8^+^ T cells.

Previous histopathological studies have indicated that melanocytes in the lesional skin diminish while the surrounding basal cells remain intact. This phenotype suggests the occurrence of apoptosis specifically in melanocytes. We co-cultured the CD8^+^ T cells from peripheral blood samples or the perilesional margin of vitiligo skin lesions with autologous melanocytes at various ratios. The results revealed that CD8^+^ T cells from different sources induced apoptosis of autologous melanocytes. However, the perilesional CD8^+^ T cells induced more extensive apoptosis in the autologous melanocytes, suggesting higher cytotoxicity. The reason may be that T cells infiltrating the perilesional margin specifically recognize melanocyte antigen and cause melanocyte-specific killing effects, while T cells from peripheral blood only cause non-specific killing. Taken together, our data further support the theory that CD8^+^ T-cell infiltration is the mechanism of vitiligo pathogenesis.

To further investigate the killing mechanism of CD8^+^ T cells on autologous melanocytes, we examined the cytokine levels in the supernatants of CD8^+^ T cells and autologous co-culture using cytokine arrays. The results revealed that IL-6 and IL-13 levels were the highest in the perilesional CD8^+^ T cell/melanocyte co-culture. This confirms the previous observations that IL-6 and IL-8 levels are elevated in the sera of vitiligo patients while the TNF-α and IFN-γ levels are decreased (18), and that IL-6 is highly produced in the vitiligo lesional skin (19–21).

IL-16 is a multi-functional cytokine which is involved in the regulation of immune response, hematopoiesis, acute phase reaction and inflammation (22,23). IL-6 has been reported to significantly upregulate the expression of ICAM-1, an adhesion molecule among melanocytes which is a potent growth inhibitor of melanocytes (24–26) and has been proven to be necessary for lymphocytes to adhere to and induce immune injury in melanocytes (21). IL-6 itself contributes to the immune injury of melanocytes by enhancing the migration of effector cells and the adhesion between effector cells and target cells. IL-6 downregulates the expression of microphthalmia transcription factor (MITF) and its downstream genes that cause the shrinkage of melanocytes and suppress melanin synthesis. The production of IL-6 is significantly increased by IL-17A which is secreted by Th17 cells (27).

Furthermore, IL-6 affects the antibody production of B cells (28,29), and induces the differentiation of Th17 (30–32) and cytotoxic T cells (33). Accumulating evidence suggests that IL-6 is closely related to various autoimmune diseases, including rheumatoid arthritis, Crohn’s disease, multiple myeloma and systemic lupus erythematosus. IL-6 levels are significantly elevated in the blood and the local microenvironment of vitiligo patients (27). Further study is required to elucidate how IL-6 is involved in the killing effect of infiltrating CD8^+^ T cells on melanocytes.

In our study, we found that IL-13 is produced by CD8^+^ T lymphocytes. IL-13, predominantly secreted by active T cells, is the immune regulator of several cell types. Anti-CD28 antibody is demonstrated to induce the transcription of IL-13 mRNA, cause the chemotaxis of monocytes, prolong the survival time of monocytes *ex vivo* and inhibit the induction of monocytes/macrophages or inflammatory factors, including IL-1, IL-6 IL-8 and TNF-α by LPS. IL-13 is one of the main regulators of allergic inflammation (34) and the key inducer of several type-2 cytokine-dependent pathologies (35,36). It functions during the pathogenesis and prognosis of certain skin disorders. However, its relevance to the occurrence of vitiligo has not been reported. The role of IL-13 in vitiligo remains to be determined.

In summary, our data demonstrate that CD8^+^ T cells from perilesional region of the vitiligo-affected skin induce autologous melanocyte apoptosis leading to the disappearance of melanocytes. Our data suggest that IL-6 and IL-13 are important in the cytotoxicity of CD8^+^ T lymphocytes to autologous melanocytes.

## Figures and Tables

**Table I:** Expression levels of CD69 and CD137 in CD8^+^ T cells from 10 vitiligo patients (mean ± SD).

Marker	Peripheral blood	Perilesional margin	t-value	p-value
CD69	29.93±2.35	56.13±3.55	6.154	<0.0001
CD137	25.97±1.63	41.74±1.06	4.550	0.0002
